# Covid-19 pandemic impacts on follow-up of child growth and development

**DOI:** 10.11606/s1518-8787.2022056004279

**Published:** 2022-06-20

**Authors:** Gisele Nepomuceno de Andrade, Leonardo Ferreira Matoso, Tércia Moreira Ribeiro da Silva, Mark Anthony Beinner, Márcia Christina Caetano Romano, Ed Wilson Rodrigues Vieira

**Affiliations:** I Universidade Federal de Minas Gerais Escola de Enfermagem Programa de Pós-Graduação em Enfermagem Belo Horizonte MG Brasil Universidade Federal de Minas Gerais. Escola de Enfermagem. Programa de Pós-Graduação em Enfermagem. Belo Horizonte, MG, Brasil; II Universidade Federal de Minas Gerais Escola de Enfermagem Departamento de Enfermagem Materno-Infantil e Saúde Pública Belo Horizonte MG Brasil Universidade Federal de Minas Gerais. Escola de Enfermagem. Departamento de Enfermagem Materno-Infantil e Saúde Pública.Belo Horizonte, MG, Brasil; III Universidade Federal de São João Del-Rei Programa de Pós-Graduação Mestrado Acadêmico Enfermagem Divinópolis MG Brasil Universidade Federal de São João Del-Rei. Programa de Pós-Graduação Mestrado Acadêmico Enfermagem. Divinópolis, MG, Brasil

**Keywords:** Infant Welfare, prevention & control, Child Health Services, Maternal-Child Health Services, Facilities and Services Utilization, COVID-19

## Abstract

**OBJECTIVE:**

To analyze the impact of the covid-19 pandemic on the use of primary health care services to follow-up the child growth and development in Brazil.

**METHOD:**

A total of 7.9 million consultations of children (0–2 years old) across Brazil between March 2017 and May 2020 were studied. Differences between medians were analyzed using non-parametric tests, the Global Moran Index (I_GM_) and the Local Indicators of Spatial Association.

**RESULTS:**

During the initial period of the pandemic, the median number of consultations was significantly lower than the same period in previous years, reducing more than 50%. The drop in 2020, compared to 2019, ranged from 49% to 62.2% across all regions of the country, except the South. The percentage reduction registered in 2019–2020 showed significant spatial autocorrelation (I_GM_ = 0.20; p = 0.04), with the presence of low-low (states with reduction between 29% and 51%) and high-high (states with reduction between 55% and 69%) spatial clusters.

**CONCLUSION:**

The covid-19 pandemic impacted the follow-up of child growth and development in primary health care services in Brazil, with a geographically uneven reduction.

## INTRODUCTION

Severe acute respiratory syndrome coronavirus-2 (SARS-CoV-2), the etiologic agent of the coronavirus disease 2019 (covid-19), is the third coronavirus responsible for severe respiratory and systemic infections, resulting in high mortality rates over the past two decades, being the first to cause a pandemic^1^. In February 2020, the first case of covid-19 was reported in Brazil. Currently, in mid-January 2022, the country registered more than 23 million cases and 621,000 deaths, being the second country with more deaths from covid-19 in the world, only behind the United States^4^.

Although the definitive impacts of the pandemic on health systems remain unclear, many countries have already shown consequences, especially a reduction in elective care services and reduced rates of individual clinical care for children in primary care services^1,5^. The pandemic also compromised strategies for the child growth and development (CGD) follow-up service, such as nutritional care and home visits^9,10^.

Political-organizational and public health factors and individual decisions contributed to this impact on the use of services. From an individual perspective, fear of the disease has a strong impact on the intentions to seek care and, consequently, on the use of health services^6,8^. From a political-organizational and public health perspective, measures against the spread of the virus or the need to invest resources in dealing with critical needs due to coronavirus infections have discouraged the use of health care, including regular follow-up of the growth and development of children in primary health care (PHC) services^11^. The inaccessibility to health services because of the pandemic is also a reality in other countries^12^.

Primary health care routine consultations aimed at following-up child growth and development were crucial to reduce child mortality since the Brazilian health reform^13^. Since the 1980s, surveillance of growth and development has been one of the main strategies of the national child health care policy^14^. Therefore, the damage caused by the cancellation or postponement of child health care is undeniable. In Brazil, situations such as food insecurity and decline in vaccination coverage have been reported^15,16^.

Although the reduction in the use of services to follow-up child growth and development in primary health care in Brazil is foreseeable, the dimension of this impact and its regional distribution is still uncertain. We aimed to analyze the impact of the covid-19 pandemic on the use of PHC services to follow-up the child growth and development in Brazil.

## METHODS

This was a descriptive and analytical study, with a cross-sectional ecological design, using the data from the *Sistema de Informação em Saúde para a Atenção Básica* (SISAB – National Primary Health Care Information System) of the Brazilian Ministry of Health, during March, April and May 2017–2019 (before the pandemic) and the same months in 2020 (beginning of the pandemic). These first three months were studied because they represent a phase of uncertainties about how control measures could affect the routine of health care service. Since 2013, SISAB has been the information system responsible for collecting data for the Brazilian PHC services, which is part of the *Estratégia e-SUS Atenção Básica* (e-SUS AB – e-SUS Primary Care strategy)^17^. The year 2017 was chosen because it represents the beginning of an increase in the adherence of PHC teams regarding the transmission of health data information to the SISAB database, when compared to previous years^18^. The data are available at https://sisab.saude.gov.br/.

In order to extract the data, all consultations provided to follow-up the growth and development to children up to two years old in the studied periods in the states were considered. The data generated information regarding the follow-up of growth and development that represent elective and periodic care, focused on children health promotion, disease prevention, early detection and rehabilitation^14^. Since the source information system for the data is restricted to the public health system, the data does not refer to the entire population of the country. However, the public system is responsible for providing health care to approximately 70% of the Brazilian population, showing the representativeness of the data studied^19^.

The units of analysis were the regions and states of the country. The regions (North, Northeast, Southeast, South and Center-West) configure state groupings, following the Brazilian territorial division. The highest Human Development Index (HDI) is in the Southeast region, while the lowest is in the North and Northeast regions. The Midwest region has the second highest HDI, practically tied with the South region^20^. The infant mortality rate, a global indicator of development, is lower in the South region, with 10.1 deaths per thousand live births, and it is higher in the North region, with 15.4 deaths. The Southeast, Midwest and Northeast regions had rates of 11.3, 11.6 and 14.1 deaths per thousand live births, respectively^21^.

In order to analyze the number of visits over time (2017–2020), the difference between their medians was considered. The medians of each region and the country were compared in this timeframe, for each Region and for the country. The percentage reduction of the median of the number of visits to the health services to follow-up the child growth and development, per year, compared to 2020, was estimated using the equation [(median number of visits in the year - median number of visits in 2020) / median number of visits in the year × 100]. The data were tabulated and analyzed using the Statistical Package for the Social Sciences software (IBM-SPSS, v.19, IBM, Chicago, IL).

To analyze the spatial distribution of the reduction in the median number of visits among states, the Moran Global Index (Moran I) and the Local Indicator of Spatial Association (LISA) were used^22^. Moran I is was an indicator that measured the spatial autocorrelation of the reduction in the median of visits using a measure ranging from -1 to +1.

The spatial autocorrelation will be negative if the states with a similar reduction in the median of visits are far from each other (they will not form clusters). On the other hand, the spatial autocorrelation will be positive if the states with a similar reduction in the median of visits are close to each other (they will form clusters). To interpret the strength of a spatial correlation, Moran I was classified as weak (< 0.3), moderate (0.3–0.7) or strong (> 0.7) and the significance level considered was 0.05^22^. Regarding LISA, patterns of reduction distributions on the medians of visits among states were analyzed: high-high reduction (states with high reduction surrounded by states with high reduction) and low-low reduction (states with low reduction surrounded by states with low reduction). TerraView software (version 4.1.0) was used for spatial analysis and QGIS software (version 3.18.3) was used for spatial representation.

The Moran Local Index was calculated to verify whether states that have similar reductions in the number of visits are close to each other, forming clusters, or if they are randomly distributed, with no pattern of occurrence.

This study uses data from the public domain, with open and unrestricted access, and without identifying individuals. The assessment was waived by the research ethics committee of the *Universidade Federal de Minas Gerais* (UFMG) (CAAE: 46914221.5.0000.5149).

## RESULTS

This study analyzed 7,952,396 child growth and development (CGD) health consultations conducted across the country between March 2017 and May 2020. We observed an increase in the median number of health consultations between 2017 and 2019, and a drastic drop in 2020. In this last year, during the beginning of the pandemic, the median number of visits was lower than the median number of visits conducted over the same period of all previous years, with a reduction of more than 50% (Table 1).

**Table 1:** Median variation, interquartile range and percentage reduction of the median number of child growth and development consultations performed with children aged 0 to 2 years old, between March and May, from 2017 to 2020, in Brazil.

Year	Median (P25 e P75)	Reduction of the median toward 2020 (%)
2017	57,444 (19,050–90,918)	51
2018	65,182 (23,288–108,280)	57
2019	67,973 (22,294–110,214)	59
2020	28,148 (12,921–61,195)	Ref.

When analyzing the data by regions, a significant difference was observed for the medians of the number of visits between 2017 and 2020, except for the Midwest region. There was a decrease in the median number of CGD visits in 2020, compared to 2019. The Northeast region demonstrated the most significant impact, with the highest percentage of reduction (62.6%). On the other hand, the South region had the lowest percentage of reduction (Table 2).

**Table 2:** Median variation in the 2017–2020 period, interquartile range and percentage reduction of the median between 2019–2020, according to the number of child growth and development consultations performed with children aged 0 to 2 years old by region, between March and May, from 2017 to 2020, in Brazil.

	Median (P25-P75)				Reduction 2019–2020 (%)
	2017	2018	2019	2020	
N	10,330 (3,967–34,153)	11,978 (5,472–45,621)	14,369 (5,858–46,012)	7,119 (2,588–22,875)	50.4
NE	67,644 (53,423–138,445)	77,783 (59,727–151,476)	76,176 (60,499–157,991)	28,765 (24,668–54,096)	62.2
SE	178,457 (56,994–286,323)	207,300 (79,547–383,360)	187,681 (68,684–453,031)	91,185 (27,239–277,857)	51.4
MW	22,826 (4,770–41,692)	31,241 (23,139–42,060)	39,330 (25,764–43,311)	20,045 (14,611–26,213)	49.0
S	77,946 (62,446–79,464)	92,024 (78,251–106,357)	94,272 (80,087–109,086)	61,195 (44,694–63,880)	35.1

N: North; NE: Northeast; SE: Southeast; MW: Midwest; S: South.

During the 2019–2020 period, the percentage reduction in the number of consultations resulted in a significant and yet weak spatial autocorrelation (Moran I = 0.20; p = 0.04), with the presence of low-low and high-high spatial clusters (Figure). Low-low type clusters (reduction between 29% and 51%) were observed in the states of Acre and Tocantins (North region), Minas Gerais and São Paulo (Southeast region), Paraná (South region), Goiás, Mato Grosso and Mato Grosso do Sul (Midwest region). High-high type clusters (reduction between 55% and 69%) were observed in the states of Ceará, Rio Grande do Norte, Paraíba, Pernambuco, Alagoas, Sergipe and Bahia (Northeast region), Espírito Santo and Rio de Janeiro (Southeast region), Amapá and Pará (North region).

**Figure:**
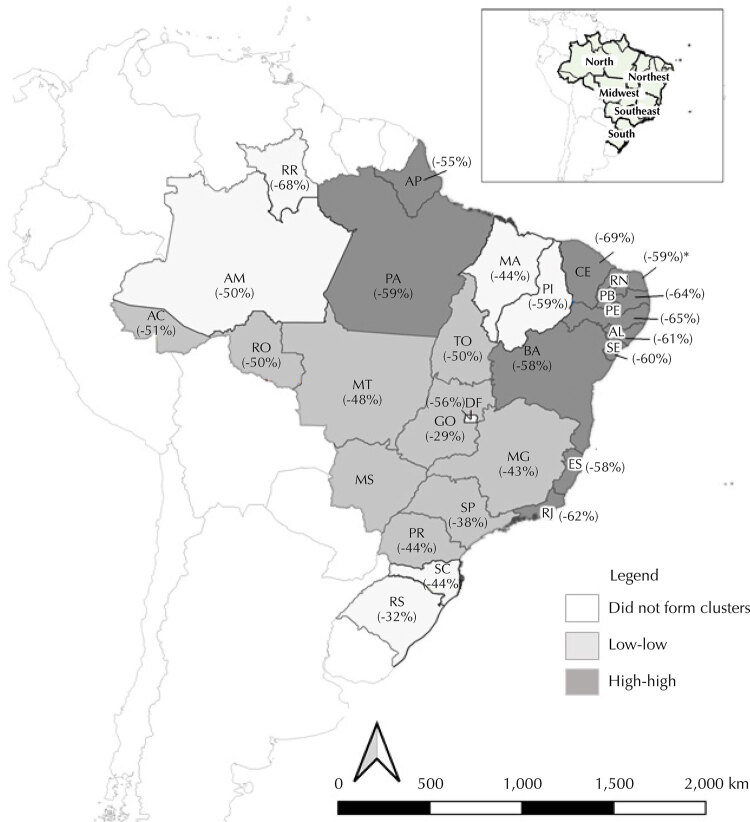
Map of Local Indicators of Spatial Association (LISA) according to the percentage reduction in the number of child growth and development consultations performed with children aged 0 to 2 years old, between March and May, from 2019 to 2020, in Brazil.

## DISCUSSION

During the first months of the covid-19 pandemic, the use of PHC services for CGD was significantly lower than that recorded in previous years in Brazil. The North and Northeast regions of the country reported reductions compared to the same period in 2019. Spatial clusters, formed by groups of states with higher or lower percentages of reduction in CGD visits, were identified.

A reduction was expected because the restrictions of elective care was a key aspect to control the pandemic^11^. In other countries where restricting non-urgent care was a strategy, the use of preventive services by children were also reduced, but less than what we observed in Brazil. During the first three months of the pandemic, the rate of well-child visits in 431 primary care clinics in the United States declined 47%^8^. Many follow-up programs changed capacity, the timing, frequency or content of clinical assessments, compromising their ability to ascertain infants’ medical and developmental needs^23^. In Qatar, the use of well-baby services reduced 40% in May and returned to numbers higher than expected in June^12^.

The geographic differences of the impact of the pandemic on CGD follow-up were found either by analyzing the medians of the numbers of visits by regions or by the spatial analysis of states. The Northeast region was the most impacted, considering the highest percentage of reduction and the highest number of states in one high-high cluster. The differences in investments in the health sector in the Northeast region and the challenges imposed by the covid-19 pandemic combined for the impact on children’s access to health services in the region^24,25^.

Regarding the regional differences in the reduction of CGD visits, recent studies assessed children’s low vaccination coverage during the covid-19 pandemic in Brazil, with great impact in the Northeast region compared to the South^16,26^. This result reflects the incidence and mortality rates from covid-19. In the analyzed period, the Northeast and South regions showed an incidence rate above the national mean^27^. Besides, states with a high reduction of CGD visits also had very high rates of incidence and death from covid-19 over the same study period, such as Amapá, Pará, Ceará, Pernambuco and Rio de Janeiro^27^. Therefore, the higher incidence and mortality rates, due to covid-19, may have contributed to a substantial drop in the number of visits by healthy children and regular check-ups. These results are concerning, since the sharpest reductions occurred in regions that have showed the highest historical infant mortality rates^13,28^.

To analyze territorial aspects, we must consider the interdependence and inseparability of political, economic and geographic aspects between the Brazilian regions, since Brazil’s territorial extension has many regional differences in terms of politics and economy^29^.

The significant drop in the number of CGD visits may determine negative impacts on mortality indicators^30^. Similarly, the interruption of services that provide routine maternal and child care may compromise other health indicators, such as immunization data^16^. The reduction in the number of CGD routine visits can increase food insecurity, because the assessment of nutritional status, behavior and food consumption are essential aspects to follow-up CGD^31^.

It is necessary to consider that many chronic conditions in childhood are followed-up during CGD visits, by keeping regular contact with health professionals. Thus, the worsening of such conditions may occur over time, given the reduction in CGD care. Besides systematic follow-up of children with chronic conditions, CGD care enables health professionals to approach the most vulnerable children and families, favoring the early identification of conditions that compromise the full development of children^32^.

Social restrictions and health recommendations imposed new determinants on children’s health. With the significant decline in routine childcare, many of the health issues may not have received the necessary care. We also emphasize the increase in cases of violence against children during the pandemic. Because of restricted access to CGD measures, it may not have been diagnosed, since most of these diagnoses are made during routine consultation^30^. Therefore, PHC must be observed and so does the impact of the pandemic on this sector, such as mental disorders, domestic violence and the worsening of chronic conditions. There were few national investments for this level of care, which may be clear from the results of our study.

Health systems must be prepared to ensure non-face-to-face follow-up from now on, especially in a context where physical contact should be avoided. The use of telehealth is a strategy to eliminate distancing barriers. However, it requires government efforts to provide the technological and human infrastructure necessary for its implementation^33^.

During the pandemic, virtual care has gained an important incentive and may have come to stay. However, the focus of telemedicine actions in the country was aimed at combating the pandemic and not meeting other population health needs, thus not explaining the reduction observed in face-to-face visits^34^.

To stop the pandemic, extra precautions were required for the safety of children and health care professionals, for example, the restructuring of the service flow for similar demands and age groups, proper procedures for disinfection of facilities and equipment, correct use of personal protective equipment and investments for the implementation and expansion of telehealth services. Social distancing during consultations and alternative schedules, hand hygiene and proper handling of suspected cases are among the necessary adaptations. Public health services need to be better organized to meet the children’s needs and prevent them from risking their health, related to the provision of care or interruption thereof^35^.

The limitation of this study is the use of secondary data, which were not collected specifically to answer the research questions. Our study only considered data gathered according to the country’s states. Therefore, our results do not show differences between or among municipalities in the same state. Other studies that assess the impact of the pandemic at the level of municipal health systems are necessary, since health decisions in Brazil are decentralized in municipalities. Furthermore, the children’s ages at the time of the consultations were not considered, making it essential for future studies.

In conclusion, during the first three months of the covid-19 pandemic there was a negative impact with an uneven reduction in the follow-up of CGD in PHC services in the regions of Brazil, mainly in the Northeast region. The pandemic may have influenced the demand and offer of consultations aimed at following-up for growth and development of Brazilian children, thus reducing the number of consultations conducted during its initial period. Our study may help public health policymakers understand the impact of the covid-19 pandemic on PHC services and lead them to monitor CGD, design recovery plans to manage the backlog of consultations and prevent worsening of child health indicators.
